# Blended cognitive behaviour therapy for children and adolescents with mitochondrial disease targeting fatigue (PowerMe): study protocol for a multiple baseline single case experiment

**DOI:** 10.1186/s13063-021-05126-7

**Published:** 2021-03-01

**Authors:** I. L. Klein, K. F. E. van de Loo, T. J. Hoogeboom, M. C. H. Janssen, J. A. M. Smeitink, E. van der Veer, C. M. Verhaak, J. A. E. Custers

**Affiliations:** 1 Radboud university medical center, Radboud Institute for Health Sciences, Radboud Center for Mitochondrial Medicine, Department of Medical Psychology, PO Box 9101, Geert Grooteplein Zuid 10, 6500 HB Nijmegen, The Netherlands; 2 Radboud university medical center, Radboud Institute for Health Sciences, IQ Healthcare, PO Box 9101, Geert Grooteplein Zuid 10, 6500 HB Nijmegen, The Netherlands; 3 Radboud university medical center, Radboud Institute for Molecular Life Sciences, Radboud Center for Mitochondrial Medicine, Department of Internal Medicine, PO Box 9101, Geert Grooteplein Zuid 10, 6500 HB Nijmegen, The Netherlands; 4 Radboud university medical center, Radboud Institute for Molecular Life Sciences, Radboud Center for Mitochondrial Medicine, Department of Pediatrics, PO Box 9101, Geert Grooteplein Zuid 10, 6500 HB Nijmegen, The Netherlands; 5 International Mito Patients Association, Bergambacht, The Netherlands

**Keywords:** Mitochondrial disease, Fatigue, Blended cognitive behaviour therapy, Single case experiment, Children and adolescents

## Abstract

**Background:**

Mitochondrial disease is a rare, hereditary disease with a heterogeneous clinical presentation. However, fatigue is a common and burdensome complaint in children and adolescents with mitochondrial disease. No psychological intervention targeting fatigue exists for paediatric patients with a mitochondrial disease. We designed the PowerMe intervention, a blended cognitive behaviour therapy targeting fatigue in children and adolescents with mitochondrial disease. The aim of the intervention is to reduce perceived fatigue by targeting fatigue-related cognitions and behaviours.

**Methods:**

A multiple baseline single case experiment will be conducted in five children (8–12 years old) and 5 adolescents (12–18 years old) with mitochondrial disease and severe fatigue. Patients will be included in the study for 33 weeks, answering weekly questions about the fatigue. Patients will be randomly assigned a baseline period of 5 to 9 weeks before starting the PowerMe intervention. The intervention consists of face-to-face and online sessions with a therapist and a website with information and assignments. The treatment will be tailored to the individual. Each patient will work on their personalized treatment plan focusing on personally relevant goals. The primary outcome is perceived fatigue. Secondary outcomes are quality of life, school presence and physical functioning.

**Discussion:**

The results of the PowerMe study will provide information on the efficacy of a blended cognitive behaviour therapy on reducing perceived fatigue and its impact on daily life in children and adolescents with mitochondrial disease. Strengths and limitations of the study design are discussed.

**Trial registration:**

Dutch Trial Register NTR 7675. Registered on 17 December 2018. Identifier https://www.trialregister.nl/trial/7433

## Background

Mitochondrial diseases (MD) are rare inherited metabolic diseases; the vast majority are caused by defects in the oxidative phosphorylation system, the final biochemical pathway involved in cellular energy production [1]. Cells that make up organs and tissues that require a lot of energy to function properly contain a higher number of mitochondria. These high-energy organs and tissues are also most commonly affected in a MD; examples are the brain, heart, skeletal muscles and kidneys [2]. As a rule of thumb, symptoms can occur in ‘any organ or tissue, at any age and with any mode of inheritance’ [3, 4]. The severity of the symptoms can vary from mild to highly debilitating complaints. Symptoms can fluctuate over time; they can remain stable over long periods of time, or rapidly deteriorate. The course of the disease is often progressive [5].

Current treatments focus mainly on support and symptomatic relief. Most commonly used interventions include exercise and dietary supplements [5–8]. Psychological interventions may be a valuable addition to improve coping with the symptoms and reduce the impact of the disease in daily life. Psychological interventions have shown to improve symptom load, disability and school attendance in children and adolescents with various somatic complaints (e.g. functional abdominal symptoms, mixed-pain complaints, chronic fatigue syndrome) [9].

Many patients are confronted with the disease and its symptoms during childhood: in 81% of patients diagnosed with MD, onset is before the age of 18 years [10]. Fatigue is often present in children with MD and rated as one of the most burdensome complaints. Perceived fatigue can be defined as an overwhelming sense of tiredness, lack of energy and feeling of exhaustion [11]. Fatigue in patients with chronic diseases, including MD, may be influenced by certain disease-related and generic factors. Disease-related factors that contribute or cause fatigue in patients with MD are directly related to the disease, and could include the mitochondria, and affected organs and tissues [12]. Several generic factors can influence fatigue through cognitions and behaviours [13, 14]. In children with MD, studies show deregulated levels of physical activity, disease-related distress and sleep disturbances [15–19]. In addition, dysfunctional cognitions and behaviours may also be present, such as all-or-nothing behaviour, fear avoidance, symptom focusing and catastrophizing [20]. Cognitions and behaviours can be targeted successfully to reduce fatigue in several chronic diseases by using cognitive behaviour therapy (CBT) [14, 21, 22].

**Fig. 1:**
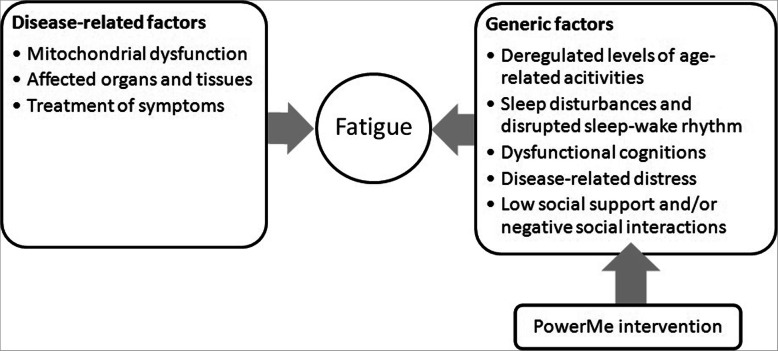
Proposed explanatory model of contributing factors to MD-related fatigue and targets of the PowerMe intervention

Many generic factors contributing to fatigue can be successfully treated with CBT. The basic components of CBT focus on the relationship between thoughts, feelings and behaviours. To our knowledge, there is no existing psychological intervention targeting severe fatigue in children and adolescents with MD. We propose a blended CBT aimed at reducing perceived fatigue: the PowerMe intervention (see Fig. 1). The intervention is based on existing theories of perpetuating factors in disease-related fatigue [13, 23]. CBT can help patients cope with the many uncertainties and limitations they experience, provide insight and tools to use their energy more optimally and explore barriers and solutions for changing their activities. Online CBT interventions seem to be effective in treating both psychiatric and somatic conditions in children and adolescents [24].

PowerMe is a combination of face-to-face CBT and an online environment to support therapeutic contact and assignments. Using a mostly online approach will limit travelling for the patients. As a result, patients can more optimally use their time and energy on the intervention [13, 23]. The PowerMe intervention is tailored to the individual patient. Each patient will work on personally relevant goals. In addition, the intervention takes into account the age-related differences in children and adolescents, the diverse clinical phenotype of MD that may cause a variety of consequences in daily life and the presence of (one or more) generic factors contributing to fatigue. All generic factors presented in Fig. 1 can be targeted in the intervention. In addition, parents will be included as an at-home coach to improve the effectiveness of the intervention [25, 26].

Although CBTs are traditionally tested using randomized controlled trials, this study design is not applicable when large groups are difficult or impossible to obtain. A study design focusing on the individual level can also be valuable. Both types of study designs provide important, useful and unique information about the effectiveness of an intervention [27]. To investigate the effect of a tailored intervention in patients with a rare disease, a within-subject design is more feasible. A multiple baseline single case experimental design is suitable, because each participant will act as his or her own control comparing data of the baseline phase with the intervention phase [27–29].

We will investigate the efficacy of the PowerMe intervention in a multiple baseline single case experiment. We hypothesize that the intervention significantly reduces perceived fatigue in severely fatigued children and adolescents with MD. Furthermore, it is hypothesized that due to the character of the intervention, a delayed effect will take place, approximately on the half-way point of the individual treatment (in most cases after 8 weeks). In addition, the efficacy of the PowerMe intervention on reducing the negative impact on daily life will be studied by measuring quality of life, school presence and physical functioning.

## Methods/design

### Study design

A multiple baseline single case experiment will be conducted to evaluate the efficacy of the PowerMe intervention on reducing perceived fatigue in children and adolescents with MD. In addition, the effect of the intervention on quality of life, school presence and physical functioning will be studied.

### Recruitment and study population

Five patients between 8 and 12 years old and five patients between 12 and 18 years old will be included. Specialized metabolic physicians and nurses working at the Radboud Center for Mitochondrial Medicine at the Radboud university medical center in the Netherlands will recruit children diagnosed with MD. Patients will be informed about the PowerMe study during regular medical follow-up, or by an information letter. Furthermore, advertisements will be placed online by the patient organization International Mito Patients (IMP) and the Dutch Foundation for Muscular Diseases (‘Prinses Beatrix Spierfonds’). All interested patients will be screened for eligibility with online questionnaires. Inclusion and exclusion criteria are shown in Table 1. Patients and their parents will sign informed consent forms before filling out the questionnaires. Patients who meet the criteria for a possible anxiety or depressive disorder will be interviewed by a psychologist to make an appropriate assessment of the eligibility of the patient for the study or whether a referral to regular care is advised. Subsequently, the researcher will contact the patient and parents with the results of the screening. If the patient is eligible for the study, the researcher will give a more detailed explanation about the study and answers all questions of the family. Informed consent will then be signed.

**Table 1:** Inclusion and exclusion criteria

Inclusion criteria	Exclusion criteria
- Age between 8 and 18 years - Able to speak, write, and read Dutch - Diagnosed with mitochondrial disease (genetically confirmed) - Being severely fatigued (CIS fatigue severity ≥35) - Access to a computer with internet connection - Basic computer skills - Able to travel to the hospital for the CBT intervention (3 sessions)	- Intellectual disability (developmental age younger than 8 years). - Primary depression (CDI ≥16) or anxiety disorder (SCARED-C ≥ 25) - Current psychological treatment for fatigue

### Procedure

To ensure sufficient data points are present to analyse the data, this multiple baseline single case experiment consists of 33 weekly measures of fatigue dispersed over three consecutive phases: baseline, treatment and follow-up. The intervention will be introduced staggered across time. In other words, each participant will start after a different length of the baseline period. The participants are allocated to an intervention start point at random. The following five start points will be randomized: the therapy will start following a baseline period of 5, 6, 7, 8 or 9 weeks. This approach is similar in both groups. The baseline period should be stable. Five to nine data points should give a clear indication of the stability of the baseline [28, 30]. However, extending the baseline period when an unstable baseline is present will cancel out the randomization. For a more rigorous method of testing the effect of the intervention, baseline randomization will be applied. Therefore, baseline stability will not be checked before starting the intervention.

The researcher will contact the patient after randomization has allocated a baseline period between 5 and 9 weeks. At the start of the study, a participant number will be assigned to the patient to ensure confidentiality of personal identifiable patient data throughout the entire study. Participants and their parents receive a link to the online questionnaires each week for 33 weeks: the children fill out assessments about their fatigue and parents report on the school presence of their child. At the start of the study, the patient and parents are asked to fill out additional online questionnaires for the baseline assessment (T0).

**Fig. 2:**
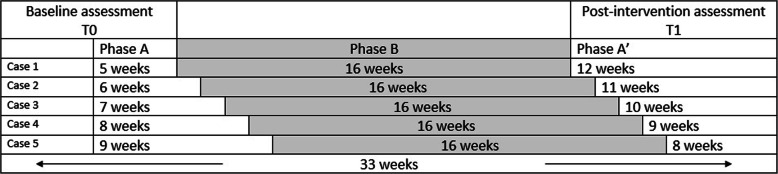
Guideline of the multiple baseline design used in the study. Note: Phase B can range from 12 to 20 weeks. Phase A′ is adapted to phase B to reach a total of 33 weekly measurements

After 5–9 weeks, the patient will start with the PowerMe treatment. The guideline for the treatment duration is 16 weeks. However, it can range from 12 to 20 weeks, tailored to the needs of each participant. Afterwards, the patient fills out the (online) post-intervention questionnaires (T1) and continues with the weekly questionnaire for 8–12 weeks. In Fig. 2, an overview of the study design is shown.

### Randomization

Unbiased assignment of the baseline period through randomization improves the internal validity of a single case experiment; it protects against readiness or eagerness to participate and rules out any bias by the researcher assigning baseline length to a participant [31, 32]. Therefore, participants will be randomly assigned to a baseline period of 5 to 9 weeks by an independent researcher [29]. Randomization of the starting point of the treatment yields statistical control over known and unknown confounding variables. In addition, the statistical conclusion validity will be enhanced by randomization [31, 33, 34]. In the proposed study, the intervention start point will be determined on a random basis using the Single-Case Data Analysis (SCDA) plug-in package for R software [35].

### Intervention

#### Development

The study and intervention were developed in collaboration with the patient organization IMP. The PowerMe website is built and designed as a healthspace in the webportal of Karify (an ehealth platform based in Utrecht, the Netherlands). The treatment protocol and modules are based on proven CBT protocols focusing on coping with fatigue and chronic illness [13, 23, 36]. Furthermore, a focus group was organized with patients, a board member of the patient organization IMP and a diverse group of health care professionals working with patients with MD. Patients’ needs and wishes for the treatment and website were mapped out and used as a guideline during the development of the blended CBT. In the focus group, patients were asked to reflect on the content, design, lay-out and feasibility of the intervention and the study. Based on their comments, certain topics were added to the intervention as (additional) content in submodules or as information in the library of the website. Furthermore, suggestions for lay-out and design were incorporated as well.

#### The treatment protocol

Trained and experienced CBT therapists and researchers collaborated to write the treatment protocol and the content of the website. The website will be used as a supportive tool within the treatment and contains information, assignments and applications for sending e-mails and making video calls in a secure environment. The website consists of six child modules and one parental module (see Table 2). The modules have been adapted to each age group: children aged 8–12 years and adolescents between 12 and 18 years old. Parents also have their own version of the website, containing all child modules and an additional parental module.

**Table 2:** The treatment modules of the PowerMe intervention

Module	Type of module	Content
‘What does your day look like?’	Obligatory	Assignments to guide the treatment plan: Two diaries will be filled out: an activity diary and a sleep diary. The patient will record all activities and rate the fatigue after the activity and enjoyment of the activity. Periods of rest will also be recorded. The sleep diary records among others bedtimes, hours of sleep and quality of the sleep. Both diaries will be filled out for 1–2 weeks. Furthermore, the patient can already write down ideas for possible treatment goals.
‘Knowledge’	Obligatory	The patient will learn more about MD, fatigue related to MD and the PowerMe intervention. It is important that the patient learns information about MD and fatigue that is reliable. Unrealistic ideas about their disease or fatigue can promote unhelpful thoughts and safety behaviours.
‘Thinking, feeling, doing’	Obligatory, including optional assignments	The main focus of this module is the relationship between thoughts, feelings and behaviours in difficult situations, and practicing helpful thoughts. Optional assignments: (1) challenging negative thoughts, (2) identifying cognitive biases and (3) a thought experiment.
‘My battery’	Obligatory, including optional assignments	Information and assignments focused on using their energy more optimally: - Activity diary: Scoring activities and finding ways to change their activities to feel less fatigued (e.g. changing very exhausting activities, different types of rest, balancing between high and low energy activities) - Working on activity-related goals using step-by-step plans - Coping with necessary and/or fun, but exhausting activities - Helping thoughts
‘Sleep’	Optional	Information and assignments about problematic sleep-wake rhythms and sleep hygiene: 1) Making and following a consistent sleep schedule keeping into account: hours of sleep during the night and bedtimes during weekdays and the weekend. 2) Changing problematic sleep hygiene/sleep habits 3) Assignments related to ruminating (thinking helping thoughts, writing down their thoughts, planning 15 min at another time to think things over, relaxation exercises)
‘Me and others’	Optional	Assignments related to communicating about their fatigue and MD. 1) Practice reactions to difficult questions or remarks, either by talking about their disease or reacting assertively. 2) Problem solving, which can focus on a wide range of problems. 3) Communicating their boundaries to help them keep balance between activities and rest.
‘After the treatment’	Obligatory	Together with the therapist, a plan will be made on how to deal with (consequences of) fatigue in the future. Also, the treatment will be evaluated. By writing down the relapse prevention plan within the PowerMe website, all information will be easily found by the patient after the treatment has ended.
‘How can I coach my child?’	Parental module	Parents have the opportunity to coach their child in daily life, whereas the therapist only has weekly contact with the patient. Tips and tricks are provided to help parents support their child during the treatment.

#### Usability testing

The website was tested by three children between 8 and 18 years old, a parent of a child with MD, a young adult with MD, two psychologists and two researchers. Their feedback on readability, usability and layout was used to optimize the final version of the website.

#### The PowerMe intervention

The intervention will be part of an interdisciplinary treatment for patients with MD. Different parts of the treatment must be tested separately before a comprehensive interdisciplinary treatment could be tested. The PowerMe intervention provides tools to help children cope more effectively with their fatigue complaints, and to experience less negative effects of the fatigue in their daily life. The intervention will be tailored by the therapist to the needs of the patient based on the results of the self-report questionnaires, the intake and related assignments. Patients will work on personally relevant goals related to their fatigue. The therapist works closely together with specialized health care professionals to ensure patients work on realistic goals and the therapist can take the possibilities of, for instance, physical activity into account for each patient. Examples of personalized goals are seeing their friends more often, distributing their energy more optimal during the day, accepting their limitations and focusing on possibilities. Additional goals may focus on coping with their disease in general and other consequences of the MD such as pain.

Patients will have three face-to-face sessions and five sessions using video calling, though this may be adapted to the needs and preferences of the patient. The dose is based on other CBT protocols targeting disease-related fatigue or distress [13, 23, 37]. In addition, the therapist will have weekly email or telephone contact with the patient to keep the patient motivated, give feedback on assignments and answer any questions.

The first intake session will be face-to-face with the patient and his/her parents. Afterwards, all patients will start recording their activities and sleep pattern in online diaries (module ‘What does your day look like?’). The second session will focus on setting relevant and realistic goals. Also, the treatment will be more elaborately explained. All participants will then continue with the ‘Knowledge’ module at home. If sleeping problems are considered important contributors to fatigue, the next session will focus on the module ‘Sleep’. This module can also be done as homework. The modules ‘Thinking, feeling, doing’ and ‘My battery’ will be discussed in the following sessions. The start of the module ‘My battery’ will be discussed in a face-to-face session, when possible. After, patients can continue the assignments by themselves or with their parents. An optional module (module 5—‘Me and others’) is given only when the child has a goal related to communication with others. The last module (‘After the treatment’) will be done in the final face-to-face session with the therapist.

Parents will also have an important role in the treatment of their child. They will coach and help their child with the treatment at home. Therefore, parents will receive information on how to coach their child and an overview of all child-modules. Parents of children between the age of 8 and 12 will most likely be present during all sessions. The therapist calls the parents at least once to discuss any questions and give personalized tips on coaching their child. If high levels of parental stress are present on the PIP or mentioned in the intake, the therapist will address these and will provide tools to help them cope with the situation. When parents require specialized help, they are encouraged to see their general practitioner. Parents can also email the therapist if they have any more questions.

### Training, supervision and treatment integrity

The intervention will be given by two healthcare psychologists licensed to treat patients with CBT and experienced in working with children with a chronic disease. The therapists will be trained by a clinical psychologist in using the PowerMe protocol and website. They will receive weekly supervision from an experienced clinical psychologist.

Treatment integrity will be evaluated by recording all sessions and other contact moments with the participant. Sessions and telephone contact will be recorded with permission of the patient and all email-contact will be saved. Treatment fidelity will be measured at the end of the study, by randomly selecting and evaluating therapist adherence to the protocol in 5% of all sessions and phone/email contacts, including the order of the sessions and content within the sessions.

### Refusal of study participation and drop-out

If patients do not wish to participate, they have the option to provide a reason. Both refusals and reasons for refusal will be recorded by the researcher. Drop-outs from the intervention are also recorded. The researcher contacts all drop-outs and records reasons for drop-outs or study-assessments that are not completed. It is not mandatory for participants to provide reasons for dropping-out. When a participant drops-out of the study, another participant will be recruited up to 5 extra participants per group.

### Adverse events

All (serious) adverse events that are reported by the participant or observed by the therapist will be recorded. During the intervention, the therapist will monitor any stress and strain that may arise due to the intervention, or caused by other circumstances, and takes appropriate action. Reported adverse events will be monitored until they have been resolved or a stable situation has been reached. Serious adverse events, as defined by the Dutch law for medical scientific research in humans, will be reported by the researcher to the Research Ethics Committee ‘CMO Region Arnhem-Nijmegen’ that approved the study protocol [38, 39].

### Outcomes

All outcome measures and data collection timepoints are listed in Table 3. A differentiation is made between parental and child questionnaires. Fatigue severity, impact of fatigue on daily life and school presence will be measured weekly. All questionnaires will be administered in Dutch.

**Table 3:** Time points of all questionnaires and other measurement instruments

	Measurements	T0	T1	Weekly	Intervention
**Main outcome measures**					
Fatigue severity	CIS, subscale fatigue severity	C	C	C	
	PedsQL-MFS	C, P	C, P		
**Secondary outcome measures**					
Quality of life	CHQ	C, P	C, P		
School attendance	School attendance questionnaire	C, P	C, P	P	
Physical functioning	CHQ, subscale physical functioning	C, P	C, P		
	PedsQL-MFS, subscale physical functioning	C	C		
Impact on daily life	Two weekly items (see the ‘Methods/design’ section)			C	
Self-rated improvement	Self-rated improvement questionnaire	C	C		
**Indicators for treatment goals and therapeutic guidance**					
Sleep problems	Sleep diary				C
Level of activity	Activity diary				C
Dysfunctional cognitions about the disease and fatigue	ICQ	C			
	Self-efficacy Scale	C			
Mental fatigue	CIS, subscale concentration	C	C		
Parenting stress	PIP	P			
**Other measures**					
Demographic variables	Age, gender, education, physical problems, and comorbidities	P			
Disease- and treatment related variables (medical record)	Diagnosis, genetic mutation, affected family members, current/past treatments, time since diagnosis				
Feasibility	Questions regarding patient eligibility, response rate, willingness to participate, commitment and satisfaction with the intervention.		C, P, T		
Evaluation/Feedback	A process evaluation focused on treatment integrity, feedback of the therapist and patient on: the intervention, use of the website, and blended nature of the intervention		C, P, T		

Note: *T0*, baseline assessment; *T1*, post-intervention assessment; *weekly*, weekly measurements throughout the entire study; *C*, child questionnaire; *P*, parent questionnaire; *T*, therapist questionnaire

#### Primary outcomes

Fatigue severity will be measured weekly using the Checklist Individual Strength (CIS) subscale fatigue severity (8-items, 7-point Likert Scale), the primary outcome measure [40, 41]. Scores range from 8 to 56; a higher score indicates a higher level of fatigue. A score of 35 or higher is an indication of severe fatigue.

In addition, fatigue severity will be measured at baseline and post-intervention assessment with the Pediatric Quality of Life Inventory Multidimensional Fatigue Scale (PedsQL-MFS) [42], and the CIS subscales ‘fatigue severity’ and ‘concentration’. Both the CIS and PedsQL-MFS have demonstrated sufficient validity, with Cronbach’s alpha’s of 0.90 and at least 0.70, respectively [40, 43].

#### Secondary outcomes

Quality of life will be measured with short forms of the Child Health Questionnaire (CHQ). The CHQ-SF for children consists of 45 items divided in 14 physical and social domains and two overall scales: physical health and psychosocial health [44]. The CHQ-CF45 is valid and reliable with a median Cronbach’s alpha of 0.89 [45]. The parent form (CHQ-PF28) is also valid; the summary measures show adequate reliability with Cronbach’s alpha larger than 0.70 [46]. Impact on daily life is also measured weekly with two items: The patient answers whether the fatigue has interfered with their activities during the week and if it did, how much distress it caused. Both items are answered on a 5-point Likert scale.

School attendance (work/internship) will be expressed in (attended hours/obliged hours × 100). Parents will answer weekly questions regarding school (work/internship) presence: how often and how long their child was not present at school, and the reasons for the absence.

Physical functioning will be measured by the subscale physical functioning of both the CHQ and PedsQL Multidimensional Fatigue Scale.

Self-rated improvement is measured by one item on which patients indicate whether they feel much better, have the same complaints or have become worse compared with the previous measurement at T0. An additional open-ended item will ask whether they consider the intervention helpful in dealing with their fatigue and if they notice a difference in daily life [26].

#### Other study parameters

Demographic characteristics will be determined during baseline assessment (T0). Disease- and treatment-related variables will be collected from the patients’ medical record via the participants’ physician.

Furthermore, the intervention will be evaluated in terms of feasibility by looking at patient eligibility, response rate, willingness to participate, commitment and satisfaction with the intervention. A process evaluation will focus on treatment integrity and feedback of the therapist and patient on the intervention, use of the website and blended nature of the intervention.

Additional instruments to assess which modules and assignments of the PowerMe intervention are indicated will be filled out during baseline assessment (T0) and consists of the following questionnaires: Illness Cognitions Questionnaire (ICQ) [47] and Self-Efficacy Scale [48, 49]. Parents will fill out a questionnaire on parenting stress related to caring for a child with a medical condition (Pediatric Inventory for Parents) [50].

### Power

The sample size and series length are based on literature of randomization tests in multiple baseline single case experiments. Power of a multiple baseline single case experiment is related to the series length [31, 33, 34]. A series length of 30 has a power over 0.8 when the effect size is 1.0 and no autocorrelation is present [33], though some autocorrelation can be expected and will be taken into account. Sample size is based on the primary outcome of fatigue severity using at least 30 weekly measurements. When using randomization tests, a sample size of at least 4 participants per group is necessary to have sufficient statistical power in the randomization test [33, 51]. Based on the literature, the current design of five patients and 33 measurements will be sufficient to reach a statistical significance *p* < .01 using the Wampold-Worsham procedure [33], with significance reached at *p* < .05.

### Intended statistical analyses

The primary objective of the PowerMe study is to examine the efficacy of the blended CBT on reducing fatigue severity in children with MD. The weekly measurements will be analysed with (1) visual analysis, (2) randomization tests and (3) multilevel modelling.

1. Visual analysis will be used for an initial assessment of the intervention effects on fatigue severity by examining six features of graphed SCE data: level, variability, trend, immediacy of effect, overlap and consistency of data patterns [52]. On a group level, heterogeneity between individual trajectories will be explored and described.
2. Randomization tests will be used to test if there is a statistical significant effect of CBT on fatigue. These tests have the ability to detect smaller changes than visual analysis alone and test statistical significance. We expect the fatigue severity to be lower in the treatment phase than in the baseline phase. Therefore, we will use a one-tailed alternative hypothesis, measuring the difference in fatigue severity between phases A and B [34, 51].
3. Multilevel modelling will be used to investigate statistical significance and treatment effects using a multiple, single case multilevel modelling strategy based on the modelling suggestions by Moeyaert et al. (2014). This approach can identify case-specific treatment effects and estimated differences between cases. The fluctuations of the weekly and/or aggregated scores between the phases (baseline, intervention and follow-up) are evaluated. More specifically, the analysis focuses on the differences in the intercept and slopes of the weekly scores from one phase to the other. This method has also been validated in small sample sizes (with *N* of 4–8) [53].

All other outcomes will be mapped descriptively. The software program RadQuest will be used to administer questionnaires, which will prevent missing data within questionnaires. According to the study design, missing measurement moments will be handled as missing data and will not be imputated. Analyses of the data will follow the intention-to-treat principle, using the SCDA plug-in package for R software [35, 54].

## Discussion

This paper outlines the study protocol for a multiple baseline single case experiment to investigate the efficacy of a blended CBT on reducing perceived fatigue and the negative impact of fatigue on daily life in children with MD. Fatigue is highly prevalent in children with MD and rated as one of the most burdensome complaints [15]. Other studies show that CBT can effectively reduce fatigue in patients with chronic disease [21, 22]. However, few evidence-based interventions exist for children with fatigue due to a chronic disease [9, 55]. Furthermore, no studies have been found targeting fatigue with a CBT intervention in patients with MD.

The blended care approach of the PowerMe intervention could be a promising working method within specialized care of rare diseases to reach patients country-wide. Travelling is limited, increasing time and energy spend on the intervention and reducing school absence. The tailored approach ensures patients work on individually relevant goals with a personalized treatment plan. Possible fatigue maintaining factors are addressed throughout the intervention. The effect of this individualized intervention will then be measured for each participant using a single case experiment.

The study design has several strengths and limitations. A single case experiment is used to measure the efficacy of an intervention in small groups. It is especially useful in rare diseases like MD, when large sample sizes are not feasible. Moreover, a single case experiment measures the effect of the intervention on an individual level. This corresponds well when investigating the individually tailored PowerMe intervention in a heterogeneous group of MD patients. Thirdly, fatigue will be measured weekly over a course of 33 weeks. The course of fatigue will be measured over time instead of measuring the difference between two data points (before and after treatment). Though this provides valuable information about the course of fatigue over time, the participant needs to commit to the study for a relatively long time. Furthermore, by focusing on perceived fatigue as primary outcome, measured with a validated self-report questionnaire, the risk of response shift bias increases. Though this is the most optimal and accurate way of measuring perceived fatigue, we need to be aware of such a bias when reporting the results of our study. Some difficulties are inherent to this design. Randomization and stable baselines are both important to improve validity. Unfortunately, controlling for a stable baseline within a randomized multiple baseline design is not possible. In addition, a single case design often presumes an immediate effect when a treatment is started. However, a psychological intervention needs time to change unhelpful thoughts and behaviours before an effect can be measured. A delayed effect can be investigated with single case designs, although it is not as straightforward as immediate effects. Regarding the statistical analyses, caution is warranted regarding the power calculation of the randomization test. A large effect size is needed to have sufficient power for this analysis, which may not be a realistic effect size for this type of intervention and outcome. However, large effect sizes have been reported in studies focusing on CBT treatments and literature suggests larger effect sizes are likely in single case designs that focus on established interventions [56, 57]. The PowerMe intervention implements widely used psychological techniques, tailored to children and adolescents with MD. We have also added multilevel modelling as an additional statistical analysis, which can be used in small groups and are more likely to detect changes when smaller effect sizes are present. Lastly, to control for effects of time and other possible confounders, it is recommended to use a concurrent design [28, 29]. However, patients may not be able to start at the same time and therapists may have limited time to treat several patients at the same time. If a concurrent design is not feasible, a non-concurrent design will be used in one or both groups to investigate the effectiveness of the intervention.

In conclusion, fatigue is highly prevalent in children with MD, a rare disease with a highly heterogeneous course and expression. Evidence-based interventions are needed to reduce fatigue and improve quality of life. This study aims to investigate the efficacy of a blended CBT to reduce perceived fatigue in children with MD using a single case experimental design. If the PowerMe intervention shows to be effective and feasible in this study, it could be a valuable addition in clinical practice to treat fatigue in children with MD. However, more research is needed to be able to provide sufficient evidence about the effectiveness of the PowerMe intervention on experienced fatigue [28]. If proven effective, the intervention will be part of an interdisciplinary treatment for patients with MD. Patients with MD often receive many different health care interventions to treat their symptoms. Future research should explore the effectiveness of a combination of treatments, for example, physiotherapy and psychological interventions, to treat fatigue on a mental and physical level.

## Trial status

Recruitment of the PowerMe study is ongoing. The recruitment started in Febuary 2019 and is expected to end in Febuary 2021.

## Data Availability

Data will be made available upon reasonable request.

## Abbreviations

CBT: Cognitive behaviour therapy
CDI: Children’s Depression Inventory
CHQ: Child Health Questionnaire
CIS: Checklist Individual Strength
ICQ: Illness Cognitions Questionnaire
IMP: International Mito Patients
MD: Mitochondrial disease
PedsQL-MFS: Pediatric Quality of Life Inventory Multidimensional Fatigue Scale
SCARED: Screen for Child Anxiety Related Disorders
SCDA: Single-Case Data Analysis
